# Knowledge, attitudes, and practices regarding traditional Chinese medicine therapies among COPD patients: a cross-sectional study

**DOI:** 10.3389/fpubh.2026.1744988

**Published:** 2026-02-19

**Authors:** Xu Yang, Ting Wen

**Affiliations:** Chengdu Xinjin District Hospital of Traditional Chinese Medicine, Department of Respiratory Medicine, Chengdu, China

**Keywords:** attitude, cross-sectional study, knowledge, practice, pulmonary disease, chronic obstructive, traditional Chinese medicine

## Abstract

**Introduction:**

This study aimed to assess the knowledge, attitudes, and practices (KAP) regarding Traditional Chinese Medicine (TCM) therapies among COPD patients.

**Methods:**

A cross-sectional study was conducted at Xinjin District Hospital of Traditional Chinese Medicine, Pidu District Hospital of Traditional Chinese Medicine, and Shuangliu District Hospital of Traditional Chinese Medicine in Chengdu, from January 11 and April 22, 2025. Data were collected using a structured questionnaire, which included demographic information and KAP-related assessments.

**Results:**

A total of 490 valid responses were obtained, yielding a valid response rate of 87.19%. Most respondents were male (67.1%), married (89.6%), and residing in rural areas (55.9%). Their mean knowledge, attitude, and practice scores were 8.89 ± 5.39 (possible range: 0–22), 39.84 ± 4.16 (possible range: 11–55), and 37.44 ± 5.68 (possible range: 11–55), respectively. The structural equation modeling results showed that the direct effect of knowledge on both attitude (β = 0.316, *P* = 0.012) and practice (β = 0.753, *P* = 0.013).

**Conclusion:**

Although participants demonstrated insufficient knowledge, generally positive attitudes, and suboptimal engagement in TCM-related practices for COPD. This gap among the three KAP dimensions underscores the need for targeted educational interventions to better align knowledge, attitudes, and practices related to TCM therapies for COPD.

## Introduction

Chronic obstructive pulmonary disease (COPD) is a leading global cause of morbidity and mortality, disproportionately affecting low - and middle-income countries, with approximately 212 million people affected and 3.3 million deaths annually (1, 2). In China, the epidemiological data reveal a particularly concerning pattern, with COPD prevalence reaching 13.7% among individuals aged 40 years and older, encompassing nearly 100 million patients and positioning COPD among the nation's three leading causes of death (3, 4). In response to this heavy burden, alternative and integrative approaches such as Traditional Chinese Medicine (TCM) have attracted growing interest in China.

TCM has demonstrated a well-established history in managing chronic respiratory disorders and is gaining increasing recognition as a valuable complementary therapeutic approach for COPD management (5). Within TCM theoretical frameworks, COPD manifests through syndromes conceptualized as “Fei Zhang” (lung distention) and “Chuan Zheng” (dyspnea), pathophysiologically attributed to lung qi deficiency coupled with phlegm retention (6). The therapeutic armamentarium of TCM encompasses diverse modalities including herbal decoctions, acupuncture, moxibustion, and acupoint application, all designed to regulate qi circulation, resolve phlegm accumulation, and restore optimal organ function (7, 8). Accumulating clinical evidence demonstrates that the integration of TCM with conventional Western medicine yields significant improvements in pulmonary function parameters, reduces exacerbation frequency, and enhances overall quality of life among patients with stable COPD (7, 9). Recent controlled trials have provided further validation of TCM's therapeutic potential, demonstrating that acupuncture intervention can effectively decelerate FEV_1_% decline (10), while specific herbal formulations have shown efficacy in improving pulmonary artery pressure and exercise capacity in COPD patients complicated by pulmonary hypertension (8).

Despite these promising therapeutic advances, the effectiveness of TCM interventions is fundamentally contingent upon patients' comprehension, acceptance, and active engagement with treatment protocols, factors that critically influence adherence patterns and ultimate clinical outcomes. The KAP framework provides a well-established methodology for evaluating individual understanding and behavioral responses to health interventions, operating on the foundational principle that knowledge acquisition shapes attitudinal formation, which subsequently influences behavioral manifestation (11, 12). Within COPD research contexts, existing KAP investigations have predominantly concentrated on patients' understanding of symptomatology, medication adherence behaviors, inhaler technique proficiency, and smoking cessation practices (13, 14). Conversely, there remains a significant knowledge gap regarding patient understanding of TCM principles and practices in managing COPD through complementary therapeutic approaches. In geographic contexts such as Chengdu, where TCM practices are deeply embedded within cultural healthcare traditions, comprehensive assessment of KAP among patients becomes essential for identifying barriers to effective TCM utilization and developing targeted, family-centered intervention strategies that enhance disease management outcomes. Therefore, this study aimed to assess the KAP regarding TCM therapies among COPD patients.

## Materials and methods

### Study design and participants

A cross-sectional study was conducted at Xinjin District Hospital of Traditional Chinese Medicine, Pidu District Hospital of Traditional Chinese Medicine and Shuangliu District Hospital of Traditional Chinese Medicine in Chengdu, from January 11 and April 22, 2025. Eligible patients were those attending the Department of Pulmonary Diseases at public TCM hospitals, with a confirmed clinical diagnosis of stable or acute exacerbation stage COPD based on the Guidelines for the Diagnosis and Treatment of Chronic Obstructive Pulmonary Disease (15). Inclusion criteria: (1) Patients aged ≥ 18 years, able to communicate effectively, and diagnosed with stable or acute exacerbation stage COPD; (2) patients receiving at least one form of TCM therapy (e.g., Chinese herbal decoction, acupuncture, acupoint application, or breathing exercises) within the past six months. Exclusion criteria: (1) Patients with severe psychiatric conditions, cognitive disorders (e.g., Alzheimer's disease), or impairments preventing completion of the questionnaire. (2) Patients with serious comorbid organic diseases (e.g., malignancies, end-stage heart failure) significantly affecting quality of life or study outcomes. The study was approved by the Clinical Research Management Committee of Xinjin District Hospital of Traditional Chinese Medicine (Approval No.: KY202518). All participants were informed about the study protocol and provided written informed consent to participate in the study. I confirm that all methods were performed in accordance with the relevant guidelines. All procedures were performed in accordance with the ethical standards laid down in the 1964 Declaration of Helsinki and its later amendments.

### Questionnaire introduction and quality control

The questionnaire design was based on the best evidence summary of TCM therapies among COPD healthcare professionals (16, 17). A pilot test was conducted with 78 respondents, of whom 77 provided valid responses after excluding one questionnaire lacking informed consent. The overall Cronbach's α coefficient for the pilot version was 0.925, with subscale reliability coefficients of 0.950 for the knowledge dimension, 0.924 for the attitude dimension, and 0.900 for the practice dimension, indicating excellent internal consistency.

The final version of the questionnaire, administered in Chinese, consisted of four sections: demographic information, knowledge, attitude, and practice dimensions. The knowledge section included 11 items, each scored as follows: “very familiar” = 2 points, “heard of” = 1 point, and “not clear” = 0 points, resulting in a total possible score ranging from 0 to 22. The attitude section comprised 11 items assessed using a five-point Likert scale ranging from “strongly disagree” (1 point) to “strongly agree” (5 points), yielding a total score range of 11–55. Similarly, the practice section consisted of 11 items rated on a five-point Likert scale from “never” (1 point) to “always” (5 points), also with a total score range of 11–55. The final questionnaire was in Chinese (a version translated into English was attached as an **Appendix**). In accordance with established KAP evaluation standards (18), scores equal to or exceeding 70% of the maximum possible score in each dimension were considered indicative of adequate knowledge, positive attitude, and proactive practice, respectively.

COPD patients who met the inclusion and exclusion criteria were invited by the research team to complete a questionnaire online after obtaining their consent. Each study patient completes the questionnaire once, with all questions being mandatory to answer. Questionnaire administration was conducted via the secure online platform “Questionnaire Star.” The questionnaire must be completed in its entirety before submission, ensuring that every question is answered 100%.

### Sample size

Sample size was calculated using the formula for a cross-sectional study: α = 0.05, *n* = (Z_(1–α/2)/δ)^∧^2 × p × (1–p), where Z_(1–α/2) = 1.96 when α = 0.05. The assumed degree of variability of *p* = 0.5 maximizes the required sample size, and δ represents the admissible error, which was set at 5% in this study. The theoretical minimum sample size was calculated to be 480, including an additional 20% to account for potential subject loss during the study.

### Statistical analysis

Data analysis was performed using IBM SPSS Statistics version 27.0 (IBM Corp., Armonk, NY, USA). Continuous variables were summarized as mean ± standard deviation (SD), while categorical variables were presented as number and percentage (*n*, %). Prior to group comparisons, all continuous variables were subjected to normality testing. For variables that conformed to a normal distribution, parametric methods such as t-tests or analysis of variance (ANOVA) were employed. For variables not conforming to a normal distribution, non-parametric methods were employed: the Mann–Whitney U-test was used for comparisons between two groups, and the Kruskal–Wallis H test was applied when comparing three or more groups. Spearman's rank correlation coefficient was calculated to assess the strength and direction of associations among knowledge (K), attitude (A), and practice (P) scores. To further identify independent factors associated with proactive practice, univariate logistic regression analyses were initially conducted, followed by multivariate logistic regression modeling using variables with a *P*-value < 0.05 in the univariate analysis. To perform logistic regression, the continuous practice score (range: 11–55) was dichotomized, where a score ≥70% of the maximum (i.e., ≥38.5 points) was classified as “proactive practice”, and scores below this threshold were classified as non-proactive practice (18). In addition, structural equation modeling (SEM) was employed to explore the interrelationships between the knowledge, attitude, and practice dimensions. While logistic regression was used to examine the independent association of candidate predictors with a dichotomized practice outcome, SEM was applied to test the hypothesized KAP pathway model and to estimate the unique direct and indirect effects among the continuous KAP dimensions within a single integrated framework. It was hypothesized that knowledge directly affects attitude and practice, while attitude directly affects practice. Model fit was assessed using multiple indices, including the root mean square error of approximation (RMSEA < 0.08), incremental fit index (IFI > 0.80), Tucker–Lewis index (TLI > 0.80), and comparative fit index (CFI > 0.80) as commonly accepted thresholds for acceptable fit. A two-sided *P*-value < 0.05 was considered indicative of statistical significance.

## Results

### Demographic information on participants

Initially, this study collected a total of 562 questionnaires. The following samples were excluded: (1) 1 case declined to participate in the study; (2) 1 case with abnormal age values; (3) 69 cases with overly short response times (less than 60 seconds); (4) 60 cases answered trap questions incorrectly. As a result, 490 valid questionnaires were included, yielding a valid response rate of 87.19%. This study included 490 COPD patients from Chengdu TCM hospitals. Key demographic characteristics and KAP scores are summarized in Table 1.

**Table 1:** Demographic characteristics and KAP scores.

**Variables**	***N* (%)**	**Knowledge, mean ±SD**	***P***	**Attitude, mean ±SD**	***P***	**Practice, mean ±SD**	***P***
*N* = 490	**–**	8.89 ± 5.39	–	39.84 ± 4.16	–	37.44 ± 5.68	–
**Gender**			0.765	**–**	0.24	**–**	0.887
Male	329 (67.14)	8.83 ± 5.26	**–**	39.69 ± 4.23	**–**	37.36 ± 5.84	**–**
Female	161 (32.86)	9.01 ± 5.66	**–**	40.14 ± 4.01		37.61 ± 5.34	**–**
Age	69.18 ± 14.19	–	–	–	–	–	–
**Residence**			< 0.001	–	0.019	–	0.027
Rural	274 (55.92)	7.69 ± 5.37	**–**	39.38 ± 4.16	**–**	37.08 ± 5.15	**–**
Urban	179 (36.53)	11.24 ± 4.34	**–**	40.46 ± 4.12	**–**	38.21 ± 6.23	**–**
Suburban	37 (7.55)	6.41 ± 6.1	**–**	40.16 ± 4.08	**–**	36.41 ± 6.29	**–**
**Education**			< 0.001	**–**	< 0.001	**–**	0.033
Junior high school and below	281 (57.35)	7.32 ± 5.3	**–**	39.33 ± 4.11	**–**	37.15 ± 5.1	**–**
High school/Vocational school	173 (35.31)	11.18 ±4.49	**–**	40.84 ± 3.97	**–**	38.29 ± 6.02	**–**
Associate/bachelor's degree and above	36 (7.35)	10.14 ± 5.91	**–**	38.97 ± 4.66	**–**	35.64 ± 7.53	**–**
**Employment status**			0.999	**–**	< 0.001	**–**	0.039
Employed	92 (18.78)	8.86 ± 5.92	**–**	38.3 ± 4.1	**–**	36.13 ± 6.83	**–**
Unemployed	398 (81.22)	8.9 ± 5.26	**–**	40.19 ± 4.1	**–**	37.74 ±5.34	**–**
**Income**			< 0.001	**–**	< 0.001	**–**	< 0.001
< 5000	255 (52.04)	5.86 ± 5.35	**–**	38.74 ± 4.05	**–**	35.84 ± 5.04	**–**
5000–10000	202 (41.22)	12.15 ± 2.9	**–**	41.22 ± 3.97	**–**	39.25 ± 5.72	**–**
>10000	33 (6.73)	12.36 ± 3.26	**–**	39.85 ± 3.73	**–**	38.76 ± 6.49	**–**
**Marital status**			0.415	**–**	< 0.001	**–**	0.885
Married	439 (89.59)	8.78 ± 5.49	**–**	40.08 ± 4.1	**–**	37.46 ± 5.49	**–**
Single	51 (10.41)	9.86 ± 4.36	**–**	37.78 ± 4.14	**–**	37.27 ± 7.11	**–**
**Duration of COPD in the Patient**			0.05	**–**	< 0.001	**–**	0.227
Less than 1 year	36 (7.35)	8.56 ± 5.63	**–**	36.89 ± 4.1	**–**	35.22 ± 7.02	**–**
1–3 years	97 (19.8)	9.65 ± 5.06		37.88 ± 4.15		37.1 ± 5.91	
3–5 years	154 (31.43)	9.49 ± 5.5		41.96 ± 3.39		37.61 ± 5.59	
More than 5 years	203 (41.43)	8.14 ±5.33		39.68 ± 3.91		37.87 ± 5.29	
**Pay attention to developments on TCM therapies for COPD**			< 0.001		0.301		< 0.001
Yes	247 (50.41)	11.89 ± 3.46		39.98 ± 4.39		39.48 ± 6	
No	243 (49.59)	5.84 ± 5.28		39.69 ± 3.92		35.37 ± 4.46	
**BMI**			< 0.001		< 0.001		0.002
Less than 18.5	111 (22.65)	10.86 ± 4.5		41.69 ± 3.42		38.79 ± 5.59	
18.5 23.9	319 (65.1)	7.78 ± 5.59		39.43 ± 3.99		36.85 ± 5.56	
Greater than 24.0	60 (12.24)	11.15 ± 3.93		38.55 ± 5.17		38.1 ± 6	
**Smoking regularly**			0.462		< 0.001		0.834
Yes	195 (39.8)	9.17 ± 5.2		38.87 ± 4.26		37.42 ± 6.29	
No	295 (60.2)	8.71 ± 5.51		40.48 ±3.97		37.46 ± 5.24	
**Drink alcohol regularly**			0.837		< 0.001		0.268
Yes	122 (24.9)	8.91 ± 5.24		37.78 ± 3.82		37.79 ± 6.72	
No	368 (75.1)	8.89 ± 5.44		40.52 ± 4.05		37.33 ± 5.29	

### Knowledge, attitude, and practice

The distribution of knowledge dimensions showed that the three questions with the lowest number of participants choosing the ‘Very familiar' option were ‘COPD includes both chronic bronchitis and emphysema, which often occur together. The main symptoms of COPD include persistent cough, sputum production, shortness of breath, or difficulty breathing, and these symptoms may gradually worsen over time.' (K2) with 16.73%, ‘The goals of COPD treatment are to control symptoms, reduce acute exacerbations, improve quality of life, and lower the risk of mortality.' (K5) with 17.55%, and ‘The primary risk factor for COPD is smoking, but long-term exposure to air pollution, occupational dust and chemicals, as well as smoke from household cooking and heating, can also contribute to the development of COPD.' (K3) with 20% (Supplementary Table 1).

Responses to the attitude dimension showed that 4.69% strongly agreed and 7.96% agreed that they do not support COPD patients trying TCM therapies alongside Western medicine treatments (A7). Meanwhile, 4.29% strongly agreed and 8.37% agreed that TCM therapies are not safe enough (A8) (Supplementary Table 2).

Responses to the practice dimension showed that 10.82% rarely and 5.31% never made efforts to quit to some extent even acknowledged the harms of smoking (P3), 16.94% rarely and 4.08% never participate in COPD-related TCM health education activities provided by the hospital (P11), 16.53% rarely and 2.65% never participate in TCM rehabilitation guidance courses (P7) (Supplementary Table 3).

### Correlations between KAP

Correlation analysis showed that there were significant positive correlations between knowledge and attitude (*r* = 0.279, *P* < 0.001) as well as practice (*r* = 0.490, *P* < 0.001). Also, there was a correlation between attitude and practice (*r* = 0.216, *P* < 0.001) (Table 2).

**Table 2:** Correlation analysis.

**Variables**	**Knowledge**	**Attitude**	**Practice**
Knowledge	1	–	–
Attitude	0.279 (*P* < 0.001)	1	–
Practice	0.490 (*P* < 0.001)	0.216 (*P* < 0.001)	1

### Univariate and multivariate analysis for practice

Multivariate logistic regression showed that knowledge score (*OR* = 1.194, 95% CI: [1.120–1.273], *P* < 0.001), attitude score (*OR* = 1.087, 95% CI: [1.025–1.154], *P* = 0.006), age (*OR* = 1.030, 95% CI: [1.010–1.051], *P* = 0.004), and pay attention to developments on TCM therapies for COPD (*OR* = 2.921, 95% CI: [1.763–4.838], *P* < 0.001) were independently associated with proactive practice (Table 3).

**Table 3:** Univariate/multivariate analysis of practice.

**Variables**	**Univariate logistic regression**		**Multivariate logistic regression**	
	**OR (95%CI)**	**P**	**OR (95%CI)**	**P**
Knowledge score	1.280 (1.217–1.346)	< 0.001	1.194 (1.120–1.273)	< 0.001
Attitude score	1.144 (1.088–1.203)	< 0.001	1.087 (1.025–1.154)	0.006
**Gender**				
Male	0.941 (0.640–1.382)	0.755	–	–
Female	ref	–	–	–
Age	1.027 (1.013–1.042)	< 0.001	1.030 (1.010–1.051)	0.004
**Residence**				
Rural	ref		ref	
Urban	1.882 (1.280–2.767)	0.001	0.849 (0.497–1.451)	0.549
Suburban	0.934 (0.449–1.943)	0.855	1.362 (0.517–3.591)	0.532
**Education**				
Junior high school and below	ref		ref	
High school/vocational school	1.819 (1.235–2.678)	0.002	0.817 (0.483–1.383)	0.452
Associate/bachelor's degree and above	1.089 (0.528–2.245)	0.817	0.777 (0.259–2.332)	0.652
**Employment status**				
Employed	ref	–	–	–
Unemployed	1.351 (0.839–2.175)	0.216	–	–
**Income**				
< 5,000	ref	–	ref	–
5,000–10,000	4.210 (2.818–6.289)	< 0.001	1.447 (0.840–2.492)	0.183
>10,000	4.411 (2.087–9.324)	< 0.001	2.153 (0.843–5.500)	0.109
**Marital status**				
Married	ref	–	–	–
Single	1.087 (0.603–1.960)	0.782	–	–
**Duration of COPD in the Patient**				
Less than 1 year	ref	–	–	–
1–3 years	1.528 (0.675–3.460)	0.309	–	–
3–5 years	1.616 (0.742–3.519)	0.227	–	–
More than 5 years	1.448 (0.675–3.106)	0.342	–	–
**Pay attention to developments on TCM therapies for COPD**				
Yes	5.775 (3.853–8.657)	< 0.001	2.921 (1.763–4.838)	< 0.001
No	ref	–	ref	–
**BMI**				
Less than 18.5	2.091 (1.349–3.243)	< 0.001	0.911 (0.533–1.556)	0.732
18.5 23.9	ref		ref	
Greater than 24.0	1.853 (1.062–3.236)	0.030	1.143 (0.584–2.239)	0.696
**Smoking regularly**				
Yes	1.203 (0.832–1.740)	0.327	–	–
No	ref	–	–	–
**Drink alcohol regularly**				
Yes	1.431 (0.946–2.165)	0.090	–	–
No	ref	–	–	–

### SEM analysis

**Figure 1:**
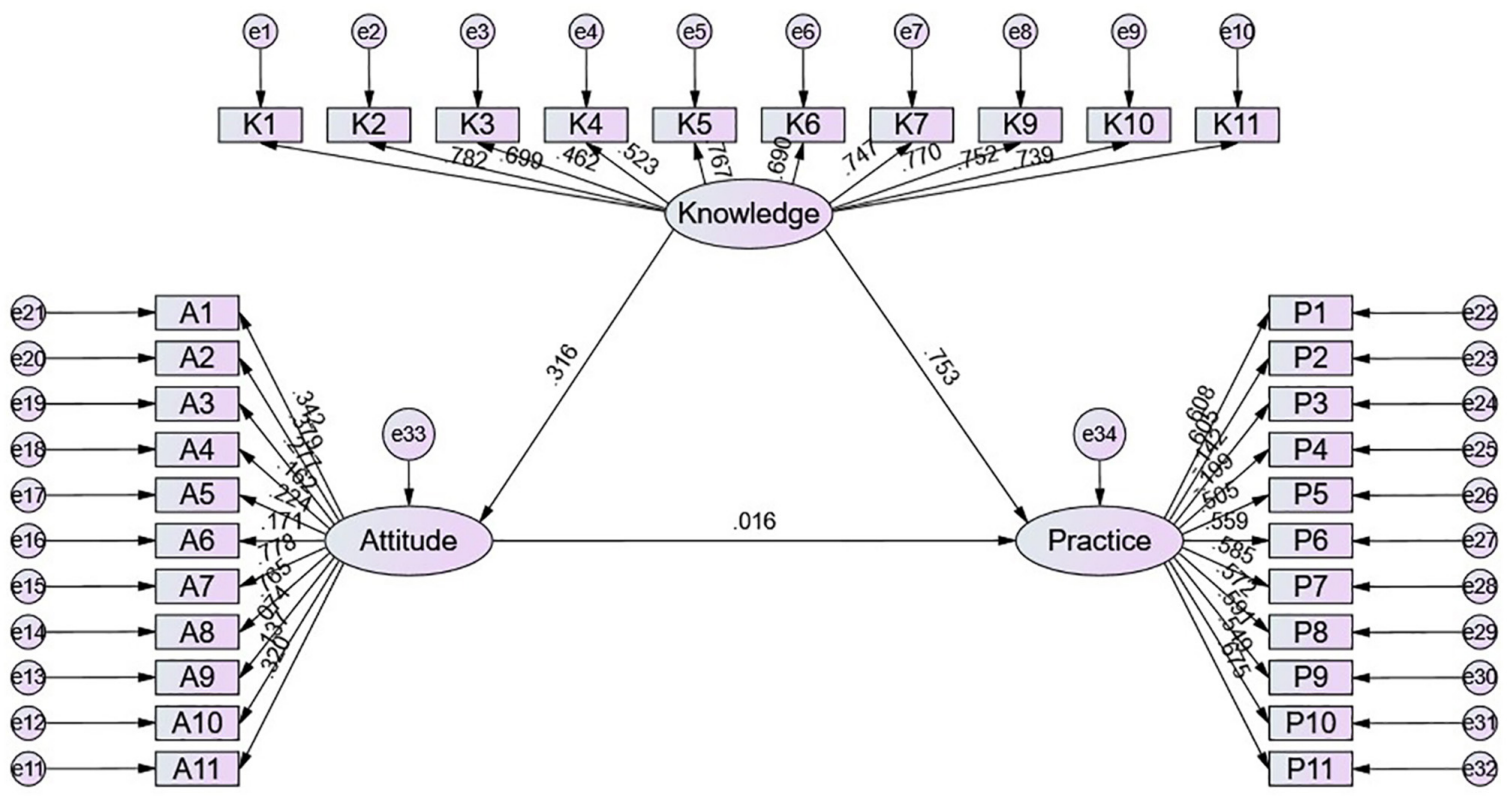
SEM model.

The fit of the SEM model yielded good indices demonstrating good model fit (*CMIN/DF* = 2.976; RMSEA = 0.064; *IFI* = 0.815; *TLI* = 0.800; *CFI* = 0.814) (Supplementary Table 4), and the effect estimates between KAP were detailed in Supplementary Table 5. The SEM results showed that the direct effect of knowledge on both attitude (β = 0.316, *P* = 0.012) and practice (β = 0.753, *P* = 0.013). However, neither the direct effect of attitude on practice nor the indirect effect of knowledge on practice was statistically significant (Table 4 and Figure 1).

**Table 4:** SEM Results.

**Model paths**	**Standardized Total effects**		**Standardized direct effects**		**Standardized indirect effects**	
	β **(95%CI)**	***P***	β **(95%CI)**	***P***	β **(95%CI)**	***P***
Knowledge → Attitude	0.316 (0.204–0.501)	0.012	0.316 (0.204–0.501)	0.012	–	–
Knowledge → Practice	0.758 (0.693–0.822)	0.012	0.753 (0.623–0.835)	0.013	–	–
Attitude → Practice	0.016 (−0.106–0.252)	0.857	0.016 (−0.106–0.252)	0.857	–	–
Knowledge → Practice	–	–	–	–	0.005 (−0.033–0.099)	0.818

## Discussion

Patients attending TCM hospitals in Chengdu exhibited insufficient knowledge, generally positive attitudes, and suboptimal engagement in practices related to TCM therapies for COPD, with knowledge showing a strong association with both attitudes and practice. Targeted educational interventions are warranted to enhance patients' knowledge, which may in turn foster more active and informed participation in TCM-based COPD management strategies. Based on the observed KAP patterns, targeted interventions should move beyond generic health education and focus on structured, practice-oriented strategies. For example, educational programs could prioritize clarifying core COPD concepts and the evidence-based role of specific TCM therapies, while incorporating practical demonstrations, repeated follow-up, and patient–family engagement to reinforce behavioral uptake. Tailoring content to patients' educational background, age, and access to healthcare resources may further enhance effectiveness.

The results of this study suggest that among patients with COPD attending TCM hospitals in Chengdu, levels of knowledge concerning both biomedical and TCM-related aspects of COPD remain modest, despite generally favorable attitudes and only partial engagement in relevant practices. This uneven distribution across the three KAP dimensions departs from conventional assumptions that view attitudes as the central mediators between knowledge and behavior. Similar results have been reported in other Chinese populations, where direct effects from knowledge to practice were stronger than those from attitudes, suggesting a bypass of evaluative reflection (14, 19). The SEM analysis in this study was grounded in the classical KAP theoretical framework, which posits that knowledge shapes attitudes and subsequently influences practices. By modeling these pathways simultaneously, SEM allowed us to examine both direct and indirect associations among the three domains while accounting for measurement error. Compared with traditional regression or pairwise correlation analyses, this approach provides a more integrated representation of the interrelationships among knowledge, attitudes, and practices, highlighting that knowledge may be directly associated with behavioral engagement even in the absence of a significant mediating role of attitudes.

Notably, the multivariate logistic regression identified attitude as a significant correlate of proactive practice, whereas the SEM path from attitude to practice was not statistically significant. This apparent discrepancy reflects differences in analytical focus and model structure. Logistic regression estimates the association between attitude and a dichotomized practice outcome while controlling for other covariates, but it does not explicitly partition shared variance among KAP domains within the hypothesized causal chain. In our data, knowledge showed a strong direct association with practice in the SEM (β*e*= 0.753, *P* = 0.013) and was also positively correlated with attitude (*r* = 0.279, *P* < 0.001). Therefore, the significant regression coefficient for attitude may partly capture variance shared with knowledge, whereas SEM evaluates the unique direct contribution of attitude to practice after simultaneously accounting for the strong knowledge → practice pathway. Under this integrated model, the incremental direct effect attributable to attitude was small and non-significant (β*i* = 0.016, *P* = 0.857), suggesting that knowledge is the primary driver of practice in this sample. Similar discrepancies between regression-based associations and SEM pathway estimates have also been reported in previous methodological and applied health research, reflecting differences in model structure and variance partitioning (20).

The knowledge domain appears constrained not only in overall score but also in the depth and specificity of understanding across key content areas. Items concerning the pathophysiological basis of COPD and the mechanisms of TCM treatment were among the least well recognized. Similar gaps in knowledge, particularly regarding the biomedical and procedural aspects of disease management, have been documented in broader surveys of Chinese adults with pulmonary conditions (21, 22).

Although bivariate correlations indicate that higher education levels are associated with greater knowledge, multivariate analyses complicate this interpretation. When controlling for income and attention to TCM-related developments, educational attainment no longer emerges as an independent predictor of behavioral engagement. This aligns with previous findings suggesting that informational access, rather than static demographic variables, more strongly influences practice behaviors (22, 23).

The structural pathway between knowledge and practice also reveals a critical point: in this population, awareness functions less as a passive reservoir of facts and more as a precondition for voluntary action. The absence of a mediating role for attitudes suggests that informational input may bypass evaluative reflection and instead operate as a direct catalyst for behavior, particularly when patients perceive TCM practices as familiar, low-risk, or culturally consonant. However, these associations should be interpreted with caution, as the cross-sectional design does not allow determination of temporal sequence or causal direction among knowledge, attitudes, and practices. Similar deviations from the conventional KAP model have been found in integrated care studies in China where familiarity and accessibility of practices, not beliefs per se, influenced uptake (14, 19).

Efforts to address these deficiencies must go beyond conventional patient education. Passive dissemination—whether through hospital leaflets or brief clinician-patient interactions—has limited reach. Community-based interventions have shown strong potential to bridge these gaps. For example, a large RCT by Lou et al. demonstrated that regular educational sessions and structured follow-up significantly improved practice adherence and reduced all-cause mortality among COPD patients (24). Similarly, an integrated community intervention in Guangdong led to a significant slowing of lung function decline and a higher smoking cessation rate over four years (25).

Despite limited knowledge, most patients expressed positive views toward TCM's role in COPD treatment. However, these attitudes, while generally supportive, lacked the depth or specificity required to meaningfully influence behavior. Other KAP studies also noted that even when attitudes are favorable, their predictive power for actual behavior remains weak, especially when cultural identity is confounded with belief strength (21, 22).

The apparent stability of these attitudes may reflect long-standing cultural familiarity with TCM rather than reflective conviction. This distinction has been supported by research showing persistent endorsement of traditional therapies among patients with low functional health literacy, independent of outcome expectations (21, 22).

Attitudinal variation across income and employment groups also warrants attention. While unemployed individuals exhibited slightly more favorable views than their employed counterparts, this difference may reflect greater familiarity with public TCM institutions rather than stronger belief in treatment efficacy. Prior studies have linked low-income preference for TCM to its lower perceived risk and accessibility rather than sustained engagement (19).

If these attitudinal patterns are to be strengthened and converted into reliable precursors of action, interventions must focus on clarifying the practical meaning of endorsement. Evidence from long-term rural interventions shows that consistent exposure to structured behavioral programs—such as guided exercise and herbal regimen phases—can significantly reduce COPD incidence and mortality (24, 26).

Of the three KAP domains, behavior scores were the most restricted. Very few respondents reported consistent adherence to recommended TCM practices, and across nearly all practice items, “sometimes” or “occasionally” was the modal response. This observation is consistent with national studies, where adherence to chronic disease self-management behaviors remains low despite general awareness and support (22). Importantly, the effect of knowledge on practice persisted after adjusting for demographic variables. Studies across respiratory and comorbid populations affirm that informational access—especially via tailored or community-based platforms—remains the most consistent predictor of action, particularly among older or rural populations (14, 23).

A more nuanced reading of the item-level data reveals further barriers. For example, items related to smoking and alcohol cessation had negative factor loadings within the SEM model, implying that these behaviors may operate independently of TCM engagement, or even in tension with it. This finding echoes broader lifestyle data, where adoption of multi-component behaviors (e.g., diet, smoking, exercise) rarely occur simultaneously among COPD patients, with only 8% practicing all three (21).

Behavioral engagement is also modulated by structural conditions. Access to consistent TCM services remains uneven, particularly for residents outside urban centers. A cluster RCT in Guangdong found that geographic accessibility significantly influenced follow-up rates and care continuity (22). Related studies point to logistical factors—transportation, appointment availability, and affordability—as major barriers to sustained engagement (19). Programs designed to enhance practice adherence must address these systemic constraints directly. From a healthcare system perspective, integrating these insights into routine COPD management may involve embedding TCM-related education into standardized clinical pathways, multidisciplinary outpatient services, and community-based follow-up programs. Collaboration between physicians, nurses, and TCM practitioners could facilitate shared decision-making and ensure that patient preferences are aligned with safe, regulated, and evidence-informed use of TCM therapies. Expanded community infrastructure, including mobile clinics and family-centered models, can extend coverage and reinforce long-term engagement, as shown in both urban and rural Chinese COPD management trials (24, 26).

These findings are consistent with broader literature on patient education and shared decision-making, which emphasizes that knowledge alone is insufficient unless it is translated into actionable guidance within supportive care environments. Prior studies have shown that structured education combined with ongoing professional support is more effective than passive information delivery in promoting sustained self-management behaviors. Within the context of traditional therapy regulation, these results further underscore the importance of guiding patient engagement through standardized, evidence-based frameworks rather than relying solely on cultural familiarity or personal beliefs.

This study has several limitations. First, its cross-sectional design precludes causal inference between knowledge, attitudes, and practices, limiting the ability to determine temporal or directional relationships. Second, the exclusive recruitment of participants from public TCM hospitals represents a form of selection bias, as patients who actively seek care in TCM settings may already hold more favorable attitudes toward traditional therapies or have prior positive experiences with TCM. As a result, the observed attitude scores (mean = 39.84) may be artificially inflated and may not be representative of the broader COPD population receiving care in general hospitals or community health centers (27). This selection pattern may also reduce variability in attitude scores, thereby limiting the statistical ability of attitude to emerge as a significant independent predictor of practice behavior in multivariable and structural models. Moreover, this bias may partly explain the observed pattern in which generally positive attitudes did not translate into corresponding levels of practical engagement, suggesting that such attitudes may reflect cultural familiarity or healthcare-seeking preferences rather than deeply internalized beliefs capable of driving sustained behavioral change (28). Third, self-reported data are subject to recall and social desirability biases, which may have influenced the accuracy of responses regarding attitudes and behavioral practices. Future studies should consider multi-center, stratified sampling strategies that include TCM hospitals, general hospitals, and community healthcare institutions to improve representativeness and to better quantify the impact of institutional context on patient attitudes and behaviors.

In conclusion, patients attending TCM hospitals in Chengdu exhibited limited knowledge, generally positive attitudes, but insufficient practical engagement with TCM therapies for COPD, highlighting a disconnect between perception and behavior. Targeted educational interventions are warranted to bridge knowledge gaps and promote evidence-based TCM practices, ultimately enhancing patient participation and optimizing integrative COPD management.

## Data Availability

The original contributions presented in the study are included in the article/supplementary material, further inquiries can be directed to the corresponding author.

## References

[1] Christenson SA Smith BM Bafadhel M Putcha N. Chronic obstructive pulmonary disease. Lancet. (2022) 399:2227–42. doi: 10.1016/S0140-6736(22)00470-6 35533707

[2] Safiri S Carson-Chahhoud K Noori M Nejadghaderi SA Sullman MJM Ahmadian Heris J . Burden of chronic obstructive pulmonary disease and its attributable risk factors in 204 countries and territories, 1990-2019: results from the Global Burden of Disease Study 2019. Bmj. (2022) 378:e069679. doi: 10.1136/bmj-2021-069679 35896191 PMC9326843

[3] Yin P Wu J Wang L Luo C Ouyang L Tang X . The burden of COPD in China and its provinces: findings from the global burden of disease study 2019. Front Public Health. (2022) 10:859499. doi: 10.3389/fpubh.2022.859499 35757649 PMC9215345

[4] Zhang X Lei Z Wu Y Song Y Wu X Yang B . Prevalence and risk factors for COPD in an urbanizing rural area in Western China: a cross-sectional study. Int J Chron Obstruct Pulmon Dis. (2023) 18:459–68. doi: 10.2147/COPD.S400213 37038543 PMC10082583

[5] Matos LC Machado JP Monteiro FJ Greten HJ. Understanding traditional Chinese medicine therapeutics: an overview of the basics and clinical applications. Healthcare. (2021) 9:257. doi: 10.3390/healthcare9030257 33804485 PMC8000828

[6] Zhang J Jiang B. Effect of traditional Chinese and western medicine combined with lung rehabilitation training on pulmonary function in patients with chronic obstructive pulmonary disease complicated with chronic cor pulmonale and evaluation of efficacy. Bratisl Lek Listy. (2023) 124:221–7. doi: 10.4149/BLL_2023_035 36598314

[7] Chan KH Tsoi YYS McCall M. The effectiveness of traditional Chinese medicine (TCM) as an adjunct treatment on stable COPD patients: a systematic review and meta-analysis. Evid Based Complement Alternat Med. (2021) 2021:5550332. doi: 10.1155/2021/5550332 34188688 PMC8195656

[8] Liang R Liu D Li H Yan Y Xie W Zhai Z. The efficacy and safety of herbal formulas for adults with pulmonary hypertension combined with chronic obstructive pulmonary disease: a systematic review and meta-analysis involving 1,865 participants. J Thorac Dis. (2024) 16:5923–35. doi: 10.21037/jtd-24-471 39444852 PMC11494573

[9] Fan S Zhang Z Wang Q. Efficacy of acupuncture therapy for stable chronic obstructive pulmonary disease: a systematic review and meta-analysis. Medicine. (2023) 102:e33537. doi: 10.1097/MD.0000000000033537 37058051 PMC10101258

[10] Xu G Luo Q Sun M Huang L Liu J Yang C . Effectiveness and safety of acupuncture as an adjunctive therapy for chronic obstructive pulmonary disease: a randomised controlled trial. BMC Complement Med Ther. (2024) 24:326. doi: 10.1186/s12906-024-04630-y 39227880 PMC11370288

[11] Khalid A Haque S Alvi S Ferdous M Genereux O Chowdhury N . Promoting health literacy about cancer screening among muslim immigrants in Canada: perspectives of imams on the role they can play in community. J Prim Care Community Health. (2022) 13:21501319211063051. doi: 10.1177/21501319211063051 35118911 PMC8819818

[12] Tahani B Asgari I Golkar S Ghorani A Hasan Zadeh Tehrani N Arezoo Moghadam F. Effectiveness of an integrated model of oral health-promoting schools in improving children's knowledge and the KAP of their parents, Iran. BMC Oral Health. (2022) 22:599. doi: 10.1186/s12903-022-02644-x 36510207 PMC9744048

[13] De Fatima De Oliveira Graca Y Yang L Mingyu C Alpona AB Watanabe T Sawada Y . Smoking attitudes, self-reported practices, and COPD knowledge among adults aged 20–59 years: insights from a Japanese sample. Tob Induc Dis. (2025) 23, 1–9. doi: 10.18332/tid/200855 40161904 PMC11951969

[14] Su L Wang L Ding J Zhang X Wang R Bai X . Knowledge, attitudes and practices regarding pulmonary rehabilitation among patients with chronic respiratory diseases: a cross-sectional questionnaire-based study in a tertiary hospital in China. BMJ Open. (2025) 15:e085944. doi: 10.1136/bmjopen-2024-085944 39842929 PMC11784421

[15] MacLeod M Papi A Contoli M Beghé B Celli BR Wedzicha JA . Chronic obstructive pulmonary disease exacerbation fundamentals: Diagnosis, treatment, prevention and disease impact. Respirology. (2021) 26:532–51. doi: 10.1111/resp.14041 33893708

[16] Cai Y Ren X Wang J Ma B Chen O. Effects of breathing exercises in patients with chronic obstructive pulmonary disease: a network meta-analysis. Arch Phys Med Rehabil. (2024) 105:558–70. doi: 10.1016/j.apmr.2023.04.014 37150427

[17] Liu W Liu XM Huang YL Yu PM Zhang XW Zhao C . Tai Chi as a complementary exercise for pulmonary rehabilitation in chronic obstructive pulmonary disease: a randomised controlled trial. Complement Ther Med. (2023) 78:102977. doi: 10.1016/j.ctim.2023.102977 37625624

[18] Lee F Suryohusodo AA. Knowledge, attitude, and practice assessment toward COVID-19 among communities in East Nusa Tenggara, Indonesia: a cross-sectional study. Front Public Health. (2022) 10:957630. doi: 10.3389/fpubh.2022.957630 36388283 PMC9659730

[19] Yu F Xu W Ma X Yang Y Gao J Yan X. Knowledge, attitudes and practices of healthcare professionals in the management of patients with hypertension and concurrent bronchial asthma: a cross-sectional study in the yellow river delta region of China. BMJ Open. (2025) 15:e088743. doi: 10.1136/bmjopen-2024-088743 39842923 PMC11784232

[20] Cheng J Feng Y Liu Z Zheng D Han H Liu N . Knowledge, attitude, and practice of patients with major depressive disorder on exercise therapy. BMC Public Health. (2024) 24:323. doi: 10.1186/s12889-024-17821-6 38287298 PMC10826117

[21] Cheng C Zhang W Jin B Yang S Lu H Ren Y. Knowledge, attitude, and practice (KAP) towards pulmonary nodules among Chinese adults: a mediation analysis. Sci Rep. (2024) 14:28950. doi: 10.1038/s41598-024-79657-9 39578539 PMC11584867

[22] Zhao L Zhao Q. Knowledge, attitude, and practice toward disease prevention among a high-risk population for chronic obstructive pulmonary disease: a cross-sectional study. Int J Nurs Sci. (2023) 10:238–44. doi: 10.1016/j.ijnss.2023.03.012 37128490 PMC10148251

[23] Qin Z Zhao X Meng Y Wu Y Qian J Yin M . Knowledge, attitudes and practices of intensive care unit physicians towards the management of acute respiratory distress syndrome in China: a cross-sectional survey. BMJ Open. (2025) 15:e092069. doi: 10.1136/bmjopen-2024-092069 39870496 PMC11772931

[24] Lou P Chen P Zhang P Yu J Wang Y Chen N . A COPD health management program in a community-based primary care setting: a randomized controlled trial. Respir Care. (2015) 60:102–12. doi: 10.4187/respcare.03420 25371402

[25] Adhikari TB Neupane D Karki A Drews A Cooper B Högman M . Community-based intervention for prevention and management of chronic obstructive pulmonary disease in Nepal (COBIN-P trial): study protocol for a cluster-randomized controlled trial. Trials. (2021) 22:1–10. doi: 10.1186/s13063-021-05447-7 34289879 PMC8293490

[26] Yuan X Tao Y Zhao JP Liu XS Xiong WN Xie JG . Long-term efficacy of a rural community-based integrated intervention for prevention and management of chronic obstructive pulmonary disease: a cluster randomized controlled trial in China's rural areas. Braz J Med Biol Res. (2015) 48:1023–31. doi: 10.1590/1414-431x20154385 26352697 PMC4671529

[27] Colacci M Loffler A Roberts SB Straus S Verma AA Razak F. Patient complexity, social factors, and hospitalization outcomes at academic and community hospitals. JAMA Netw Open. (2025) 8:e2454745. doi: 10.1001/jamanetworkopen.2024.54745 39813029 PMC11736502

[28] Timmermans I Meine M Zitron E Widdershoven J Kimman G Prevot S . The patient perspective on remote monitoring of patients with an implantable cardioverter defibrillator: Narrative review and future directions. Pacing Clin Electrophysiol. (2017) 40:826–33. doi: 10.1111/pace.13123 28612995

