# Supplementary material for: Knowledge, attitudes, and practices regarding traditional Chinese medicine therapies among COPD patients: a cross-sectional study
Source: Front Public Health. 2026 Feb 19;14:1744988. doi: 10.3389/fpubh.2026.1744988 (PMC12960584; doi:10.3389/fpubh.2026.1744988)
Supplement: Supplementary file 1 [file Supplementary_file_1.docx]

**Supplementary table 1. Distribution of knowledge dimension responses**

|  | **N (%)** | | |
| --- | --- | --- | --- |
|  | **Very familiar** | **Heard of it** | **Unclear** |
| 1. **Chronic Obstructive Pulmonary Disease (COPD) is a common chronic inflammatory lung disease characterized mainly by airflow limitation. This airflow limitation is usually progressive and associated with abnormalities in the airways and alveoli.** | 159 (32.45) | 183 (37.35) | 148 (30.2) |
| 1. **COPD includes both chronic bronchitis and emphysema, which often occur together. The main symptoms of COPD include persistent cough, sputum production, shortness of breath, or difficulty breathing, and these symptoms may gradually worsen over time.** | 82 (16.73) | 241 (49.18) | 167 (34.08) |
| 1. **The primary risk factor for COPD is smoking, but long-term exposure to air pollution, occupational dust and chemicals, as well as smoke from household cooking and heating, can also contribute to the development of COPD.** | 98 (20) | 265 (54.08) | 127 (25.92) |
| 1. **Patients with COPD should avoid exposure to tobacco smoke and other irritant gases in daily life, engage in appropriate physical activity to strengthen lung function, maintain good nutritional status, and receive regular influenza and pneumonia vaccinations to prevent respiratory infections.** | 113 (23.06) | 267 (54.49) | 110 (22.45) |
| 1. **The goals of COPD treatment are to control symptoms, reduce acute exacerbations, improve quality of life, and lower the risk of mortality.** | 86 (17.55) | 209 (42.65) | 195 (39.8) |
| 1. **Treatment options for COPD include smoking cessation, pharmacological therapy (such as bronchodilators, anti-inflammatory drugs, and corticosteroids), pulmonary rehabilitation, oxygen therapy, and, in some cases, surgical interventions. Smoking cessation is a critical preventive and therapeutic measure for COPD patients, as it can slow the further decline of lung function.** | 112 (22.86) | 199 (40.61) | 179 (36.53) |
| 1. **Traditional Chinese Medical (TCM) therapies for COPD are comprehensive regulatory approaches that tailor treatments to the patient’s specific constitution and condition. They aim to harmonize organ functions, relieve symptoms, and improve quality of life.** | 109 (22.24) | 186 (37.96) | 195 (39.8) |
| 1. **TCM treatments for COPD are based on syndrome differentiation, such as lung Qi deficiency, lung-spleen Qi deficiency, and lung-kidney Qi deficiency. Different herbal formulas are used accordingly. Common prescriptions include Yupingfeng Powder, Bufei Decoction, and Liu Jun Zi Tang. Patent medicines like Yupingfeng Granules and Maxing Granules are also frequently used.** | 131 (26.73) | 185 (37.76) | 174 (35.51) |
| 1. **Main TCM therapeutic methods for COPD include herbal medicine, acupuncture, cupping therapy, Tui Na (massage) therapy, external application of herbal medicine, herbal aerosol inhalation, acupoint application, and acupoint injection.** | 103 (21.02) | 214 (43.67) | 173 (35.31) |
| 1. **TCM herbal therapy for COPD acts through multiple targets and pathways. It can effectively relieve symptoms such as coughing and shortness of breath, improve the patient’s immunity, and generally has fewer adverse effects.** | 103 (21.02) | 216 (44.08) | 171 (34.9) |

**Supplementary table 2. Distribution of attitude dimension responses.**

|  | **N (%)** | | | | |
| --- | --- | --- | --- | --- | --- |
|  | **Strongly agree** | **Agree** | **Neutral** | **Disagree** | **Strongly disagree** |
| 1. **Do you believe in the effectiveness of Traditional Chinese Medicine (TCM) therapies in treating COPD? (P)** | 35 (7.14) | 345 (70.41) | 104 (21.22) | 4 (0.82) | 2 (0.41) |
| 1. **Do you think TCM therapies can alleviate the suffering of COPD patients? (P)** | 128 (26.12) | 180 (36.73) | 176 (35.92) | 2 (0.41) | 4 (0.82) |
| 1. **What is your attitude toward the role of TCM therapies in improving the quality of life for COPD patients? (P)** | 49 (10) | 291 (59.39) | 135 (27.55) | 11 (2.24) | 4 (0.82) |
| 1. **Do you think COPD is a serious disease that causes significant physical and psychological stress? (N)** | 177 (36.12) | 144 (29.39) | 152 (31.02) | 17 (3.47) |  |
| 1. **Do you agree that TCM therapies can be part of a comprehensive treatment plan for COPD? (P)** | 55 (11.22) | 312 (63.67) | 112 (22.86) | 5 (1.02) | 6 (1.22) |
| 1. **What is your attitude toward the application of the TCM concept of “preventive treatment of disease” in the prevention of COPD? (P)** | 84 (17.14) | 254 (51.84) | 136 (27.76) | 13 (2.65) | 3 (0.61) |
| 1. **You do not support COPD patients trying TCM therapies alongside Western medicine treatments. (N)** | 23 (4.69) | 39 (7.96) | 165 (33.67) | 158 (32.24) | 105 (21.43) |
| 1. **You think TCM therapies are not safe enough. (N)** | 21 (4.29) | 41 (8.37) | 140 (28.57) | 183 (37.35) | 105 (21.43) |
| 1. **Do you think TCM therapies require long-term adherence to achieve better outcomes? (P)** | 137 (27.96) | 225 (45.92) | 109 (22.24) | 11 (2.24) | 8 (1.63) |
| 1. **Do you recognize the value of TCM therapies in preserving and developing traditional medicine? (P)** | 70 (14.29) | 258 (52.65) | 142 (28.98) | 17 (3.47) | 3 (0.61) |
| 1. **Do you think research and promotion of TCM therapies for COPD should be strengthened? (P)** | 73 (14.9) | 259 (52.86) | 139 (28.37) | 13 (2.65) | 6 (1.22) |

**Supplementary table 3. Distribution of practice dimension responses.**

|  | **N (%)** | | | | |
| --- | --- | --- | --- | --- | --- |
|  | **Always** | **Often** | **Sometimes** | **Rarely** | **Never** |
| 1. **Do you actively consult doctors about TCM therapies for COPD?** | 91 (18.57) | 136 (27.76) | 200 (40.82) | 54 (11.02) | 9 (1.84) |
| 1. **In daily patient care, do you consider following TCM advice (e.g., dietary adjustment, acupoint massage)?** | 62 (12.65) | 146 (29.8) | 198 (40.41) | 77 (15.71) | 7 (1.43) |
| 1. **Have you acknowledged the harms of smoking and made efforts to quit to some extent?** | 221 (45.1) | 88 (17.96) | 102 (20.82) | 53 (10.82) | 26 (5.31) |
| 1. **Have you acknowledged the harms of alcohol consumption and made efforts to quit to some extent?** | 217 (44.29) | 104 (21.22) | 93 (18.98) | 65 (13.27) | 11 (2.24) |
| 1. **Do you prepare meals for the patient according to TCM recommendations?** | 49 (10) | 116 (23.67) | 227 (46.33) | 90 (18.37) | 8 (1.63) |
| 1. **Do you help the patient perform basic TCM rehabilitation exercises (e.g., breathing exercises)?** | 47 (9.59) | 137 (27.96) | 197 (40.2) | 100 (20.41) | 9 (1.84) |
| 1. **If there are TCM rehabilitation guidance courses available, how frequently do you participate in them?** | 64 (13.06) | 108 (22.04) | 224 (45.71) | 81 (16.53) | 13 (2.65) |
| 1. **Do you or your family members regularly receive TCM treatments as prescribed?** | 48 (9.8) | 128 (26.12) | 234 (47.76) | 72 (14.69) | 8 (1.63) |
| 1. **Do you regularly take the patient to see a TCM doctor for health conditioning?** | 44 (8.98) | 128 (26.12) | 232 (47.35) | 76 (15.51) | 10 (2.04) |
| 1. **Does the patient take Chinese medicine on time according to medical instructions?** | 53 (10.82) | 136 (27.76) | 217 (44.29) | 75 (15.31) | 9 (1.84) |
| 1. **How often do you participate in COPD-related TCM health education activities provided by the hospital?** | 58 (11.84) | 127 (25.92) | 202 (41.22) | 83 (16.94) | 20 (4.08) |

**Supplementary table 4. SEM fit indicators**

| **Model fit indicators** | **Ref.** | **Measured results** |
| --- | --- | --- |
| **CMIN/DF** | 1-3 excellent，3-5 good | 2.976 |
| **RMSEA** | <0.08 good | 0.064 |
| **IFI** | >0.8 good | 0.815 |
| **TLI** | >0.8 good | 0.800 |
| **CFI** | >0.8 good | 0.814 |

**Supplementary table 5. Estimated total effect coefficient**

|  |  |  | **Estimate** | **Standardized Estimate** | **S.E.** | **C.R.** | **P** |
| --- | --- | --- | --- | --- | --- | --- | --- |
| Attitude | <--- | Knowledge | 0.126 | 0.316 | 0.028 | 4.453 | <0.001 |
| Practice | <--- | Attitude | 0.039 | 0.016 | 0.109 | 0.358 | 0.720 |
| Practice | <--- | Knowledge | 0.722 | 0.753 | 0.065 | 11.124 | <0.001 |
| K1 | <--- | Knowledge | 1.000 | 0.782 |  |  |  |
| K2 | <--- | Knowledge | 0.781 | 0.699 | 0.048 | 16.223 | <0.001 |
| K3 | <--- | Knowledge | 0.504 | 0.462 | 0.050 | 10.181 | <0.001 |
| K4 | <--- | Knowledge | 0.570 | 0.523 | 0.049 | 11.643 | <0.001 |
| K5 | <--- | Knowledge | 0.897 | 0.767 | 0.049 | 18.138 | <0.001 |
| K6 | <--- | Knowledge | 0.845 | 0.690 | 0.053 | 15.954 | <0.001 |
| K7 | <--- | Knowledge | 0.926 | 0.747 | 0.053 | 17.546 | <0.001 |
| K9 | <--- | Knowledge | 0.975 | 0.770 | 0.054 | 18.224 | <0.001 |
| K10 | <--- | Knowledge | 0.895 | 0.752 | 0.051 | 17.689 | <0.001 |
| K11 | <--- | Knowledge | 0.877 | 0.739 | 0.051 | 17.328 | <0.001 |
| A11 | <--- | Attitude | 1.000 | 0.320 |  |  |  |
| A10 | <--- | Attitude | 0.416 | 0.137 | 0.167 | 2.497 | 0.013 |
| A9 | <--- | Attitude | 0.256 | 0.074 | 0.181 | 1.420 | 0.156 |
| A8 | <--- | Attitude | 3.215 | 0.765 | 0.518 | 6.212 | <0.001 |
| A7 | <--- | Attitude | 3.311 | 0.778 | 0.533 | 6.215 | <0.001 |
| A6 | <--- | Attitude | 0.525 | 0.171 | 0.174 | 3.021 | 0.003 |
| A5 | <--- | Attitude | 0.614 | 0.224 | 0.165 | 3.712 | <0.001 |
| A4 | <--- | Attitude | 0.589 | 0.162 | 0.204 | 2.891 | 0.004 |
| A3 | <--- | Attitude | 0.773 | 0.277 | 0.181 | 4.275 | <0.001 |
| A2 | <--- | Attitude | 1.273 | 0.379 | 0.251 | 5.070 | <0.001 |
| A1 | <--- | Attitude | 0.785 | 0.342 | 0.163 | 4.820 | <0.001 |
| P1 | <--- | Practice | 1.000 | 0.608 |  |  |  |
| P2 | <--- | Practice | 0.960 | 0.605 | 0.088 | 10.852 | <0.001 |
| P3 | <--- | Practice | -0.298 | -0.142 | 0.103 | -2.884 | 0.004 |
| P4 | <--- | Practice | -0.392 | -0.199 | 0.098 | -4.017 | <0.001 |
| P5 | <--- | Practice | 0.779 | 0.505 | 0.083 | 9.393 | <0.001 |
| P6 | <--- | Practice | 0.888 | 0.559 | 0.087 | 10.198 | <0.001 |
| P7 | <--- | Practice | 0.960 | 0.585 | 0.091 | 10.576 | <0.001 |
| P8 | <--- | Practice | 0.857 | 0.572 | 0.082 | 10.392 | <0.001 |
| P9 | <--- | Practice | 0.890 | 0.591 | 0.084 | 10.653 | <0.001 |
| P10 | <--- | Practice | 0.851 | 0.549 | 0.085 | 10.059 | <0.001 |
| P11 | <--- | Practice | 1.142 | 0.675 | 0.097 | 11.760 | <0.001 |
